# Construction of Electrocatalytic and Heat-Resistant Self-Supporting Electrodes for High-Performance Lithium–Sulfur Batteries

**DOI:** 10.1007/s40820-019-0313-x

**Published:** 2019-09-18

**Authors:** Xuemei Zhang, Yunhong Wei, Boya Wang, Mei Wang, Yun Zhang, Qian Wang, Hao Wu

**Affiliations:** 1 College of Materials Science and Engineering, Sichuan University, Chengdu, 610064 People’s Republic of China; 2 Department of Advanced Energy Materials, Sichuan University, Chengdu, 610064 People’s Republic of China

**Keywords:** Metal organic framework, Lithium–sulfur batteries, Cobalt sulfide, Heat-resistant, N-doped carbon foam

## Abstract

Boosting the utilization efficiency of sulfur electrodes and suppressing the “shuttle effect” of intermediate polysulfides remain the critical challenge for high-performance lithium–sulfur batteries (LSBs). However, most of reported sulfur electrodes are not competent to realize the fast conversion of polysulfides into insoluble lithium sulfides when applied with high sulfur loading, as well as to mitigate the more serious shuttle effect of polysulfides, especially when worked at an elevated temperature. Herein, we reported a unique structural engineering strategy of crafting a unique hierarchical multifunctional electrode architecture constructed by rooting MOF-derived CoS_2_/carbon nanoleaf arrays (CoS_2_–CNA) into a nitrogen-rich 3D conductive scaffold (CTNF@CoS_2_–CNA) for LSBs. An accelerated electrocatalytic effect and improved polysulfide redox kinetics arising from CoS_2_–CNA were investigated. Besides, the strong capillarity effect and chemisorption of CTNF@CoS_2_–CNA to polysulfides enable high loading and efficient utilization of sulfur, thus leading to high-performance LIBs performed not only at room temperature but also up to an elevated temperature (55 °C). Even with the ultrahigh sulfur loading of 7.19 mg cm^−2^, the CTNF@CoS_2_–CNA/S cathode still exhibits high rate capacity at 55 °C.

**Electronic supplementary material:**

The online version of this article (10.1007/s40820-019-0313-x) contains supplementary material, which is available to authorized users.

## Highlights

- Flexible hierarchical electrode architecture was constructed by rooting MOF-derived CoS_2_/carbon nanoleaf arrays into a nitrogen-rich 3D conductive scaffold.
- An accelerated electrocatalytic effect and improved polysulfide redox kinetics arising from the structure advantages were investigated.
- As-prepared composite delivers a significantly improved electrochemical performance not only at room temperature but also at elevated temperature.

**Electronic supplementary material:**

The online version of this article (10.1007/s40820-019-0313-x) contains supplementary material, which is available to authorized users.

## Graphical abstract

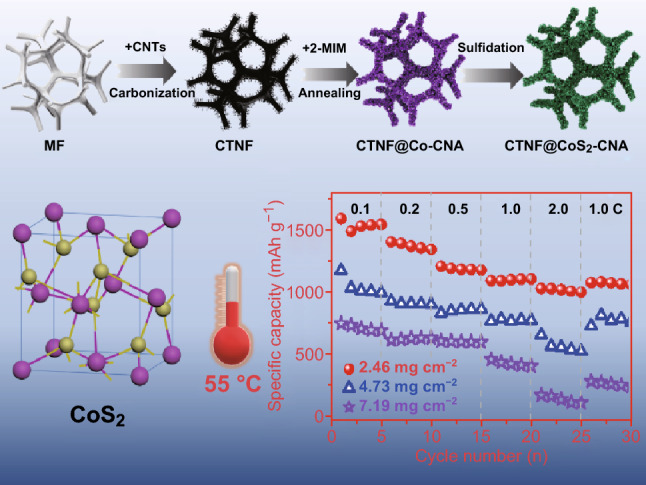

## Introduction

Currently, the demands of low-cost, renewable, and high-performance energy storage devices are exponentially increasing by the extensive growth of portable electronics and electric vehicles. In the past two decades, rechargeable lithium-ion batteries (LIBs) have prevailed and used in many fields consequently, because of a series of merits, such as large power density, long cycling stability, and slow self-discharge [1–4]. Nevertheless, considering the limits of low energy density as well as specific capacity, traditional LIBs hardly satisfy the rapidly growing demands of portable electric devices and electric vehicles. In view of this, lithium–sulfur batteries (LSBs) stand out from the competition for its outstanding merits of high theoretical capacity (~ 1675 mAh g^−1^), high energy density (~ 2600 Wh kg^−1^), abundant resources of sulfur, and environmental friendliness [5–8]. However, the commercialization of LSBs is hindered by some awkward obstacles, especially the dissolution of polysulfides (Li_2_S_*x*_, 4 ≤ *x* ≤ 8) in ether-based electrolyte, resulting in inferior Coulombic efficiency and poor cycle life [9–11]. Relatively large volumetric expansion (around 80%) during lithiation also causes the mastication of sulfur cathode and rapid attenuation of capacity [12–14]. In addition, the insulating nature of sulfur and lithium polysulfides always results in poor conductivity and irreversible loss of active sulfur, restricting the rate capability and the sulfur utilization [15, 16].

To deal with these issues, tremendous efforts have been attempted to optimize the composition and the structure of the sulfur cathode in recent years. Designing suitable host materials, applying functional interlayers, or protecting lithium anodes have been considered as effective approaches to greatly improve the electrochemical performance of LSBs [17, 18]. Porous carbon materials attract the attention of researchers to a great degree due to its excellent electronic conductivity, confined nanospace, high specific area, and high structural stability [19]. Porous carbon matrix not only facilitate charge transfer kinetics during electrochemical process, but also alleviate the migration of polysulfides through physical adsorption, as well as tolerate the volume change during lithiation/delithiation process [20–22]. Nonetheless, the interaction between nonpolar carbonaceous materials and polar sulfur species is not forceful enough to restrict the polysulfide dissolution. In light of this insight, various types of polar materials on carbon-based materials have been investigated to increase the interaction between lithium polysulfides and the electrode. Among them, transition metallic oxides like TiO_2_ [23, 24], VO_*x*_ [25, 26], and MnO_2_ [27] have been reported earlier as host materials due to its strong affinity to lithium polysulfides. However, the poor electrical conductivity of these oxides easily retards the electrode kinetics, which is unfavorable to improving the rate capability. Owing to high conductivity, electrocatalytic activity, and strong chemical interaction for lithium polysulfides, transition metal sulfides (Co_9_S_8_ [28, 29], NiS_2_ [30], and WS_2_ [31]) have been proposed as ideal sulfur-hosted materials. Rapid surface reactions arising from the electrocatalytically active components contribute to improving the sulfur utilization and alleviate the shuttle effect of soluble polysulfide intermediates [32–34]. Moreover, traditional sulfur cathodes are mostly prepared by mixing slurry and coating on metallic foils, which cannot be directly utilized as flexible batteries because it is prone to crack and exfoliate, and the coating preparation method also reduces the energy density and degrades long-term cycling performance. Furthermore, most of the reported studies only focused on the electrochemical performance of LSBs at room temperature, but there have been few attempts on the investigation of rate and cycling performance of LSBs under high-temperature environment, since the heat-resistant LSBs are actually of importance for most of the electronic equipments, especially electric vehicles (EV) and hybrid electric vehicles (HEVs).

Herein, a structural engineering strategy is employed to craft a unique hierarchical multifunctional electrode architecture constructed by rooting MOF-derived CoS_2_/carbon nanoleaf arrays (CoS_2_–CNA) into a nitrogen-rich 3D conductive carbon scaffold (CTNF@CoS_2_–CNA) to be used in LSBs. The high conductive CoS_2_–CNA combined with CNTs-wrapped N-doped carbon foam (CTNF) skeleton can form a double conductive skeleton which can availably remedy the low electrical conductivity of MOF (less than 10^−10^ S cm^−1^). Moreover, the existence of CoS_2_–CNA can greatly strengthen the chemical entrapment and promote the electrocatalytic conversion of lithium polysulfides during reaction process, further improving the active sulfur utilization efficiently and enhancing the electrochemical reaction kinetics. In addition, the interconnected conductive framework formed from 3D foam with an open structure could efficiently accelerate the electrolyte infiltration and the electron transportation. With the synergistic effect of rooted CoS_2_–CNA, in-suit nitrogen doping, and flexible 3D conductive structure, the as-prepared CTNF@CoS_2_–CNA hybrid scaffold after sulfur loading exhibits remarkable electrochemical performance when acted as flexible LSB cathode. More importantly, even worked at an elevated temperature (55 °C), high rate capability and long cycling stability which are associated with excellent heat resistance can still be achieved. The unique 3D porous structure also puts forward a worthwhile design conception to encapsulate into flexible and foldable devices for LSBs, possessing great potential for other energy storage applications.

## Experimental Sections

### Materials Preparation

#### Preparation of Flexible CTNF

Firstly, a few pieces of cleaned commercial melamine foam (noted as MF) were immersed in an aqueous CNTs suspension with a certain concentration. After repeatedly compressing and loosening MF to adsorb completely, together with drying at 100 °C for 12 h, the CTNF was finally obtained by calcined at 800 °C for 1 h with a heating rate of 5 °C min^−1^ in Ar atmosphere. Then, black CTNF was cut into the desired disk-shaped slices with a diameter of 12 mm for the subsequent experiment.

#### Preparation of ZIF-67 Precursor and CTNF@ZIF-67 Hybrid

According to the typical preparation method reported previously [35], the ZIF-67 precursor was synthesized by co-precipitation of 2-methylimidazole with divalent cobalt in deionized water. 40 mL of 2-methylimidazole (0.4 M) and Co(NO_3_)_2_·6H_2_O (0.05 M) was quickly mixed and vigorously stirred for around 10 min at room temperature. After that, the as-prepared CTNF slices were immersed into the mixed solution. After aging for 8 h, the as-prepared CTNF@ZIF-67 hybrid was collected by thoroughly washed with deionized water and dehydrated at 80 °C oven overnight. For comparison, the pure ZIF-67 precursor was prepared through the same procedure with the absence of CTNF slices.

#### Preparation of Flexible CTNF@Co–CNA and CTNF@CoS_2_–CNA Composites

The CTNF@Co–CNA was produced by annealing as-obtained CTNF@ZIF-67 slices at 800 °C for 2 h at a ramp rate of 2 °C min^−1^ under a mixture of H_2_ (5%, volume fraction) and Ar. After that, the intermediate product (CTNF@Co–CNA) was mixed with sulfur powder and calcined at 300 °C for 2 h to obtain CTNF@CoS_2_–CNA. The redundant sulfur was wiped off by calcining at 250 °C for 1 h under Ar atmosphere.

#### Preparation of CTNF@CoS_2_–CNA/S Cathode

Typically, the obtained CTNF@CoS_2_–CNA was immersed into weighing bottles which contained a certain amount of S/CS_2_ mixed solution. After evaporating the CS_2_ at static condition, the as-product was heated at 155 °C for 6 h under Ar atmosphere to obtain the CTNF@CoS_2_–CNA/S composite. A series of different sulfur loading cathodes were easily obtained by changing the concentration of S/CS_2_ mixed solution. For comparison, CTNF/S and CTNF@Co–CNA/S were also prepared through the same procedure as described in the synthesis of CTNF@CoS_2_–CNA/S.

#### Preparation of Li_2_S_8_ Solution and Adsorption Test

To experimentally identify the polysulfide adsorption ability, 5 mM of Li_2_S_8_ solution was prepared by dissolving stoichiometric sulfur and Li_2_S in 10 mL mixed solvent of 1,2-dimethoxyethane/1,3-dioxolane (DME/DOL, *v*/*v* = 1:1) using LiNO_3_ as additive. Then, the mixed solution was magnetically stirred at 55 °C for 36 h in an Ar-filled glove box to yield the Li_2_S_8_ catholyte solution. Typically, the same amounts of as-prepared NCF, CTNF, CTNF@Co–CNA, and CTNF@CoS_2_-CAN hybrids were soaked in 2.0 mL of Li_2_S_8_ solution after adsorption of 12 h for UV–Vis absorption test.

#### Preparation of Li_2_S_6_ and Symmetric Cell Assembly

The electrodes were used as identical working and counter electrodes without the presence of elemental sulfur, and 50 μL electrolyte (in DME/DOL, *v*/*v* = 1:1) containing 0.25 mol L^−1^ Li_2_S_6_ and 1 M lithium bis (trifluoromethanesulfonyl) imide (LiTFSI, 99.95%, Alfa Aesar) was added into each cell. Cyclic voltammetry (CV) measurements of the symmetrical cells were performed at a scan rate of 3 mV s^−1^ between − 1.4 and 1.4 V.

### Materials Characterization

The structure and phase analyses were conducted by X-ray diffraction (XRD, Philips X’pert TROMPD, Cu Kα radiation, λ = 1.54178 Å). Raman spectroscopy was collected on a Raman spectrophotometer (Horiba Jobin–Yvon, HR800, France) with a He–Ne laser radiation 514 nm in the wavenumber range of 800-2000 cm^−1^. The morphology and microstructure of the samples were examined by field emission scanning electron microscopy (FESEM, Hitachi, S-4800, Japan) equipped with an energy-dispersive X-ray spectrometer (EDX) and field emission transmission electron microscopy (TEM, FEI, Titan Themis 200, USA). The surface elemental compositions and valences in material were identified by X-ray photoelectron spectroscopy (XPS, Escalab 250, Thermo Fischer Scientific, USA). The specific surface area was calculated by using the multipoint Brunauer–Emmett–Teller (BET) method, and pore size distribution was characterized according to the Barrett–Joyner–Halenda (BJH) model rely on a Kubo-X1000 analyzer (Beijing Builder Electronic Technology Co., Ltd). The total pore volume was calculated at a relative pressure of 0.99 (*P*/*P*_0_). Thermogravimetric analysis (TGA) was collected with a simultaneous TGA/DSC-2 instrument (METTLERTOLEDO, USA) in the temperature range of room temperature to 600 °C at a heating rate of 10 °C min^−1^ under the protection of inert gas. UV–visible absorption spectra were measured by using UV–visible absorption spectrophotometry (UV–Vis, Shimadzu UV3600). The electrical conductivity of these composites was measured at room temperature by using a ST-2258A digital four-point probe test system (Suzhou Jingge Electronic Co., Ltd).

### Electrochemical Measurements

The as-prepared CTNF/S, CTNF@Co–CNA/S, and CTNF@CoS_2_–CNA/S sulfur-hosted composites were directly used as cathodes without any other polymer binders or conductive additives. The CR2032 coin-type cells were assembled in an argon-filled glove box for electrochemical measurements using lithium foil as the counter/reference electrode and Celgard 2400 membrane as the separator, respectively. 1.0 M lithium bis(trifluoromethanesulfonyl)imide (LiTFSI, 99.95%, Alfa Aesar) dissolved in a mixed solvent of 1, 3-dioxolane/1, 2-dimethoxyethane ((DOL/DME, *v*/*v* = 1:1) with 0.1 M LiNiO_3_ additive was used as the electrolyte. The ratio of electrolyte to sulfur was controlled as 30 µL mg^−1^. The CTNF@CoS_2_–CNA/S electrode is finally flattened into a slice with a thickness of ~ 142 μm to be assembled into the CR2032 coin-type cells. Galvanostatic charge/discharge performance tests were performed by a multi-channel battery test system (Neware CT-3008 W, China) in the voltage range of 1.5–2.8 V (vs. Li/Li^+^) at different current rates. All specific capacity values of the electrodes were calculated on the basis of sulfur weight (1 C = 1675 mAh g^−1^). Cyclic voltammetry (CV) and electrochemical impedance spectroscopy (EIS) measurements were conducted on a PARSTAT multi-channel electrochemical workstation (Princeton Applied Research, PMC1000DC, USA). And CV measurement was conducted at a scan rate of 0.1 mV s^−1^ as well as EIS was carried out over a frequency range of 100 kHz to 10 MHz with an applied amplitude of 5 mV.

## Results and Discussion

### Fabrication, Structure, and Composition of the CTNF@CoS_2_–CNA Electrode

**Fig. 1:**
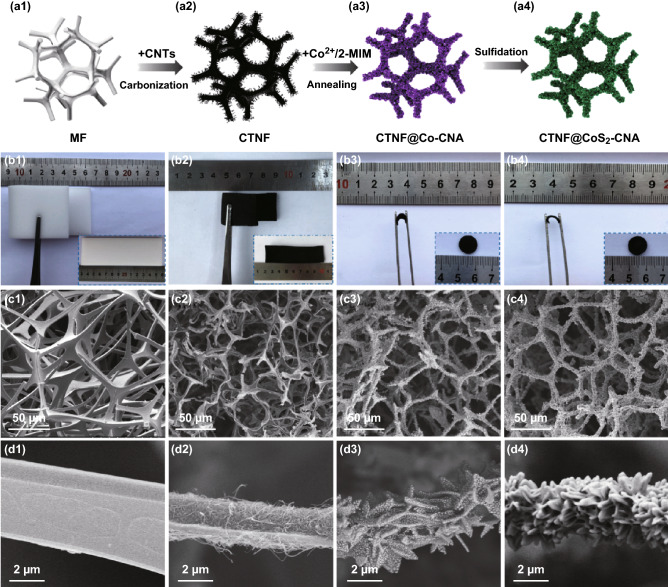
**a**_**1**_–**a**_**4**_ Schematic illustration of the synthesis process for CTNF@CoS_2_–CNA; digital photographs: **b**_**1**_ MF, **b**_**2**_ CTNF, **b**_**3**_ CTNF@Co–CNA, and **b**_**4**_ CTNF@CoS_2_–CNA; SEM images of **(c**_**1**_, **d**_**1**_) MF, (**c**_**2**_, **d**_**2**_) CTNF, (**c**_**3**_, **d**_**3**_) CTNF@Co–CNA, and (**c**_**4**_, **d**_**4**_) CTNF@CoS_2_–CNA

The crafting process of flexible CTNF@CoS_2_–CNA composite was illustrated in Fig. 1a1–a4 via a simple method, and the detailed synthetic processes were described in Sect. 2. Correlative digital photos (Fig. 1b1–b4) revealed the suppleness of all the samples, profiting from the soft feature of commercial melamine foam (MF). As being rich in the abundant N element, MF can be easy to yield a 3D N-doped carbon foam (NCF) skeleton with the high N contents of 5.16 wt% after carbonization treatment, evidenced by EDX elemental mapping images (Fig. S1). The morphology and microstructure were further investigated by SEM. MF exhibited a continuously 3D interconnected framework with smooth surface and a void size from 50 to 300 µm as shown in Fig. 1c1, d1. For the sake of ameliorating the surface roughness and conductivity, the MF was immersed into a suspension solution of CNTs to obtain the CTNF, which exhibits a considerable volume shrink after thermal polycondensation. As shown in Fig. 1c2, d2, the obtained CTNF well maintained a 3D porous carbon skeleton architecture with decent structural flexibility and robustness. Note that the shapes of MOF nanosheets depend on the self-assembled process, which were formed through the interaction between metal ions and organic ligands. Therefore, the ZIF-67 (Fig. S2) was uniformly loaded on the surface of CTNF, which served as the reactive sites for the ZIF-67 growth during the self-assembled process. The successful growth of ZIF-67 nanoleaf arrays on CTNF was further confirmed by the detection of CTNF@ZIF-67 XRD peaks (Fig. S3). Subsequently, with the thermal reduction and vulcanized treatment, the CoS_2_–CNA were rooted into the CTNF skeleton (CTNF@CoS_2_–CNA), which possesses the structure of interconnected 3D network anchored by the leaf-like nanoarray, as shown in Fig. 1c–d. The as-prepared CTNF@CoS_2_–CNA with N contents of 4.47 wt% (Fig. S4) has outstanding flexibility and mechanical strength, making itself well suited to being a free-standing electrode without conductive agents and additional binders.

**Fig. 2:**
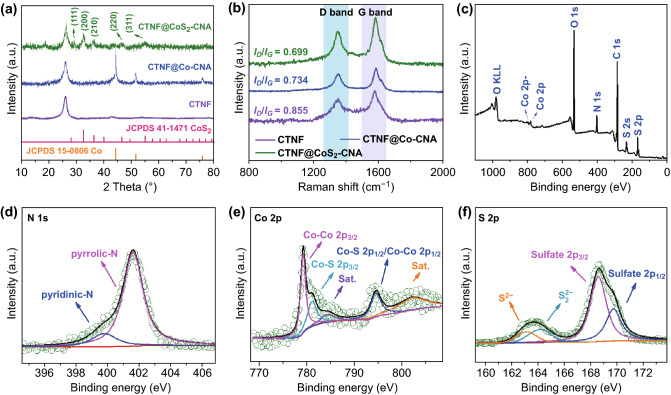
**a** XRD patterns and **b** Raman spectra of CTNF, CTNF@Co–CNA, and CTNF@CoS_2_–CNA. **c** XPS survey spectra of CTNF@CoS_2_–CNA. High-resolution XPS spectra at **d** N 1*s*, **e** Co 2*p*, and **f** S 2*p* regions of CTNF@CoS_2_–CNA composite

To compare with CTNF@CoS_2_–CNA, pristine CTNF, and CTNF@Co–CNA were also prepared as control samples. The phase and structure analyses of these samples were verified by XRD test. As shown in Fig. 2a, distinct peak at ~ 25° corresponded to the diffraction for the (002) plane of graphite was present in all the samples, indicative of a highly graphitic structure due to the high conductive of CNTs and the graphitization function of metallic Co. Additionally, CTNF@Co–CNA showed a typical diffraction pattern characteristic of Co nanoparticles (JCPDS No. 15-0806). Moreover, the diffraction peaks at 2θ values of 29.04°, 32.46°, 36.21°, 46.19°, and 54.91° can be clearly detected in the pattern of CTNF@CoS_2_–CNA, which correspond to the (111), (200), (210), (220), and (311) crystal planes of cobalt sulfide (JCPDS No. 41-1471), respectively. It is demonstrated that CoS_2_ were successfully prepared through the simple self-assembled method. Furthermore, Raman spectra were also collected to deeply investigate the composition and degree of graphitization of the materials. As depicted in Fig. 2b, two remarkable peaks are severally located at 1357 and 1591 cm^−1^, which can be assigned to defective/disorder carbon (D band) and graphitic carbon (G band), respectively [36]. The peak intensity ratio (*I*_D_/*I*_G_) of CTNF@CoS_2_–CNA (0.699) is much smaller than those of CTNF@Co–CNA (0.734) and CTNF (0.855), manifesting that CTNF@CoS_2_–CNA has a higher degree of ordered carbon structure than the others. Besides, the highly graphitic 3D carbon skeleton of CTNF@CoS_2_–CNA benefiting from the formation of CoS_2_/carbon nanoarrays also has favorable contribution on the overall electrical conductivity (Table S1), which is expected to have positive effects on the efficient pathways for electron/ion transport, thus giving rise to the amelioration in the electrochemical properties. Moreover, the conductivity of CTNF@CoS_2_–CNA is even better than most previously reported sulfur-based cathodes (Table S2).

The chemical component of CTNF@CoS_2_–CNA was analyzed by XPS measurement. The XPS survey spectra (Fig. 2c) distinctly confirmed the coexistence of C, N, Co, and S elements. The high-resolution N 1*s* spectrum (Fig. 2d) visualized the existence of two forms of N species, respectively: pyridinic-N (399.81 eV) and pyrrolic-N (401.56 eV) [37]. The existence of pyridinic N is capable to further enhance the electronic conductivity by providing various in-suit active sites which can also adsorb positively charged Li in polysulfides, thereby leading to an effective trapping of lithium polysulfides [38]. In the case of deconvoluted spectrum of Co 2*p*_3/2_ peak (Fig. 2e), two strong peaks at 779.2 and 794.5 eV derived from the 2*p*_3/2_ and 2*p*_1/2_ spin–orbit lines of CoS_2_, respectively, indicating the coexistence of Co^2+^ and Co^3+^ in CTNF@CoS_2_–CNA composite. And the peak located at 781.1 eV could be due to the Co 2*p* ion of Co–S bond. Besides, another two peaks located at around 784.4 and 802.4 eV attributed to satellite peaks (denoted as “Sat”), which can be ascribed to the Co 2*p* ion of Co–O bond [39, 40]. In case of S 2*p* spectrum (Fig. 2f), two peaks located at 168.53 and 169.69 eV were assigned to S 2*p*_3/2_ and S 2*p*_1/2_, respectively. The energy splitting value of 1.16 eV between the peak of S 2*p*_3/2_ and S 2*p*_1/2_ could be attributed to the 2*p*_3/2_ and 2*p*_1/2_ core levels of S^2−^, respectively, primarily arising from CoS_2_. All the aforementioned results proved the successful preparation of CTNF@CoS_2_–CNA for flexible self-supporting electrode materials.

**Fig. 3:**
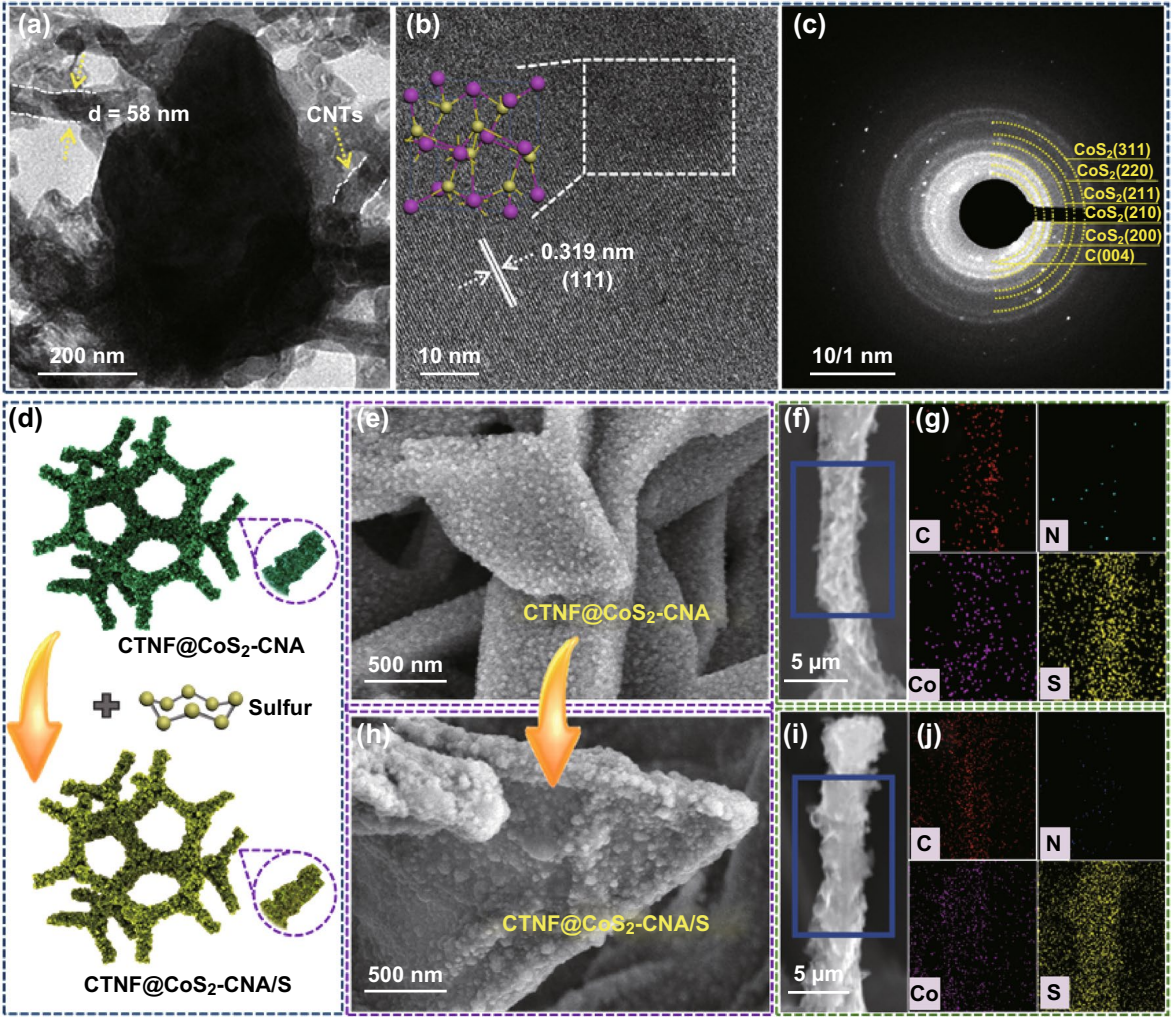
**a** TEM images and **b**–**c** High-resolution TEM images of CTNF@CoS_2_–CNA. **d** The sketch map and **e**, **h** the corresponding SEM images of sulfur-loading process. **f**, **g** Elemental mapping of C, N, Co, and S elements collected from the marked area in **f** of CTNF@CoS_2_–CNA. **i**, **j** Elemental mapping of C, N, Co, and S elements collected from the marked area in **i** of CTNF@CoS_2_–CNA/S

The detailed structure of CTNF@CoS_2_–CNA was further verified by TEM. The low magnification TEM image depicted in Fig. 3a clearly revealed that the MOF-derived CoS_2_–CNA has the configuration of leaf-like nanoflakes. Interestingly, CNTs with the diameter of 58 nm intertwining with nanoleaf can be also clearly observed, indicating the CoS_2_/carbon nanoleaf was well attached on the CNTs of CTNF, although after the ultrasonic treatment during preparation for the TEM measurement. The typical HRTEM image taken from the selected area of CoS_2_/carbon nanoleaf displayed the lattice spacing of 0.319 nm, which is corresponding to (111) crystallographic plane of face-centered cubic CoS_2_. The selected area electron diffraction (SAED) pattern (Fig. 3c) can be indexed into the CoS_2_, which was consistent with the results of XRD. Additionally, to explore the electrochemical application of CTNF@CoS_2_–CNA for LSBs, sulfur was incorporated into CTNF@CoS_2_–CNA composite via a conventional melt-infusion method (Fig. 3d). The morphology characterizations of CTNF@CoS_2_–CNA and CTNF@CoS_2_–CNA/S samples are shown in Fig. 3e–h. Note that both samples maintained the slice nanoleaf morphology and no discernible large sulfur particles exist. To further investigate the porosity of CTNF@CoS_2_–CNA and CTNF@CoS_2_–CNA/S, nitrogen adsorption/desorption isotherms were measured (Fig. S5 and Table S3). The hysteresis loop corresponding to mesopores for CTNF@CoS_2_–CNA/S is much reduced in comparison with the case for the pristine NCF@CNTs/CoS_2_ owing to the partial occupation of sulfur inside CTNF@CoS_2_–CNA, indicating that sulfur was successfully immersed into the interior cavities of CoS_2_–CNA. Furthermore, the elemental mapping evidently revealed homogeneous distribution of C, N, Co, and S elements for both CTNF@CoS_2_–CNA and CTNF@CoS_2_–CNA/S samples, ulteriorly manifesting CoS_2_/carbon nanoleaf arrays were grown on N-doped 3D conductive scaffold uniformly (Fig. 3f, g) and homogeneous sulfur dispersion (Fig. 3i, j). Such a 3D interconnected carbon network structure embedded with lithium polysulfide immobilization medium of CoS_2_ can improve the electron conductivity of the sulfur cathode (Table S1), which is favorable for enhancing the sulfur utilization and rate capability. To confirm the content of CoS_2_ in CTNF@CoS_2_–CNA hybrid and the content of loaded sulfur, TGA test was carried out. As shown in Fig. S6a, about 12.7 wt% of solid residue (Co_3_O_4_ [41]) was obtained after conducting TGA in air atmosphere, accordingly estimating the amount of CoS_2_ in the composite is around 20.9 wt%. Additionally, the sulfur content of cathode with areal sulfur-loading ~ 2 and 4 mg cm^−2^ was also verified by TGA test in N_2_ atmosphere, attesting the normal sulfur content is 38.03 wt% (~ 2 mg cm^−2^) and 51.79 wt% (~ 4 mg cm^−2^), respectively (Fig. S6b). Our work also loaded a series of different sulfur contents ranging from ~ 2 to 7 mg cm^−2^ through regulating the concentration of S/CS_2_ solution for subsequent room/high-temperature electrochemical tests.

### Electrochemical Properties at Room and Elevated Temperature

**Fig. 4:**
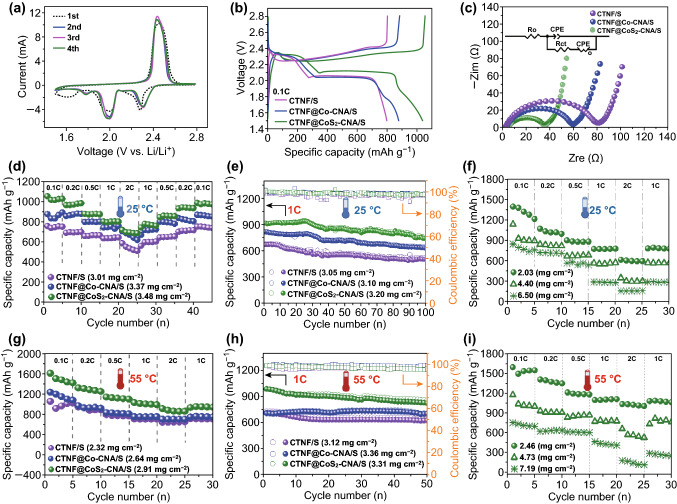
**a** CV curve of CTNF@CoS_2_–CNA/S electrode. **b** The galvanostatic charge/discharge profile of three electrodes at 0.1 C. **c** Nyquist plots of three fresh cells. Electrochemical performance comparison of three electrodes at room temperature: **d** rate and **e** cycling performance at 1 C. **f** Rate performance of CTNF@CoS_2_–CNA/S electrode with different sulfur content. Electrochemical performance comparison of three electrodes at high temperature: **g** rate and **h** cycling performance at 1C. **i** Rate performance of CTNF@CoS_2_–CNA/S electrode with different sulfur content

To evaluate electrochemical properties, the as-prepared CTNF/S, CTNF@Co–CNA/S, and CTNF@CoS_2_–CNA/S composites were directly used as cathodes, lithium foil as the anode, and a conventional PP film as the separator. Benefiting from the free-standing feature, these as-prepared composites can be directly assembled into LSBs without additional polymer binders or conductive agents, which effectively avoid the decrease of cell energy density. The electrochemical performance of these composite cathodes was characterized by cyclic voltammetry (CV) and galvanostatic charge/discharge tests in the voltage range from 1.5 to 2.8 V at 25 °C. Figure 4a shows the typical CV curves of the first four cycles of the CTNF@CoS_2_–CNA/S at a scan rate of 0.1 mV s^−1^. In the first cycle, the cathodic peak at 2.26 V corresponding to the reduction of S_8_ to soluble long-chain polysulfides (Li_2_S_*x*_, 4 ≤ *x* ≤ 8), while the cathodic peak at around 2.03 V is ascribed to the formation of insoluble short-chain polysulfides (Li_2_S/Li_2_S_2_). The broad anodic peak at 2.37 V is assigned to the reverse conversion of short-chain Li_2_S_2_/Li_2_S to S_8_. Moreover, the CV curves of subsequent cycles exhibit sharper anodic/cathodic peaks with nearly overlapped shapes after the activation of the first cycle, manifesting the high reversibility of CTNF@CoS_2_–CNA/S cathode. In addition, a broad cathodic peak located at around 1.6 V is obviously appeared at the first cycle which could be attributed to the redox reaction of CoS_2_ [42]. In order to verify this, the redox reaction of CTNF@CoS_2_–CNA matrix is also detected by CV. As shown in Fig. S7, a cathodic peak at around 1.6 V is also detected at the first cycle in the range of 1.5-2.8 V, indicating that the redox reaction of CoS_2_ is also participated in the CTNF@CoS_2_–CNA/S electrode. Meanwhile, electrochemical test was also carried out to prove this point, CTNF@CoS_2_–CNA matrix delivered a very limited discharge capacity contribution as low as around 145 mAh g^−1^ during the potential window of 1.5–2.8 V in first five cycles of 0.1 C, as well as the specific capacity greatly dropped to approximately zero in the next few laps, corresponding to galvanostatic charge/discharge curves displayed at Fig. S9. The galvanostatic charge/discharge measurements at 0.1 C were also performed with the voltage range of 1.5–2.8 V (vs. Li/Li^+^), all the profiles consist of two well-defined discharge plateaus and one charge plateau, which agree with the CV curves. As shown in Fig. 4b, the initial discharge capacity of the CTNF@CoS_2_–CNA/S is 1038 mAh g^−1^, which is higher than that of CTNF@Co–CNA/S (879 mAh g^−1^) and CTNF/S (798 mAh g^−1^). In addition, the CTNF@CoS_2_–CNA/S cathode exhibits lower polarization compared to the counter electrode (179 vs. 292 mV for CTNF@Co–CNA/S and 291 mV for CTNF/S), indicating the enhanced reactivity and better polysulfide redox kinetics for the CTNF@Co–CNA/S cathode. Moreover, the CTNF@CoS_2_–CNA/S electrode exhibits higher electrochemical performance than those of the previous reported sulfur-based electrodes (Tables S5 and S6).

EIS measurements were carried out to research the electronic/ionic transport kinetics of three cathodes. As exhibited in Fig. 4c, EIS curves of fresh cells are composed of one semicircle and one straight line. The single depressed semicircle at the high-frequency region represents the charge transfer resistance (*R*_ct_), which is an indicator of the reaction kinetics of cell [43, 44]. Accordingly, the *R*_ct_ values 77.83, 53.20, and 29.21 Ω of CTNF/S, CTNF@Co–CNA/S, and CTNF@CoS_2_–CNA/S were measured, respectively, by the equivalent circuit fitting (Table S4). Compared with the other two cathodes, the obviously decreased *R*_ct_ value indicated much faster electronic and ionic transport kinetics of CTNF@CoS_2_–CNA/S, indicating that the conductive CoS_2_ could facilitate electron and ion transfer further. Additionally, the EIS spectra after 100 cycles were also recorded (Fig. S8). The ohmic resistance of CTNF/S, CTNF@Co–CNA/S, and CTNF@CoS_2_–CNA/S after cycling increased due to some insoluble product adhered to the electrodes, while all of three cathodes showed two pressed semicircles, including one big semicircle related to the charge transfer resistance and the other inconspicuous little semicircle represents the interface contact resistance between soluble polysulfides and cathode electrode.

The application under high-temperature environment is very critical for the safety in LSBs. Designing reasonable sulfur-hosted materials not only can effectively restrict the dissolution of polysulfides into the electrolyte, but also can reduce the electrolyte consumption at elevated temperature effectively. Aim to confirm the thermostable performance, the CTNF@CoS_2_–CNA/S, CTNF@Co–CNA/S, and CTNF/S cathodes were further tested at an elevated temperature of 55 °C (Fig. 4g, h). It is seen that a remarkable discharge capacity of 1499 mAh g^−1^ at 0.1 C and 881 mAh g^−1^ at 2 C with the sulfur areal density of 2.91 mg cm^−2^ can be obtained for CTNF@CoS_2_–CNA/S, being higher than the value of the counterpart electrode and most of reported LSBs electrodes (Table 1). Besides, the CTNF@CoS_2_–CNA/S electrode emerges preferable average specific capacity of 890 mAh g^−1^ when cycled at 1 C over 50 cycles, much better than the CTNF@Co–CNA/S (723 mAh g^−1^) and CTNF/S (646 mAh g^−1^) ones. Moreover, the rate performance of CTNF@CoS_2_–CNA/S at 55 °C with a higher sulfur loading of 7.19 mg cm^−2^ is tested, which showed much higher capacities than those at room temperature because the electrochemical reaction activity of the sulfur could be expedited at a high temperature (Fig. 4i). However, with the rise of temperature, more side effects and polysulfide shuttling would occur simultaneously, as well as the material structure is more vulnerable to change, which result in the specific capacity declines rapidly and worse cycle stability (Fig. S10). Therefore, the morphology of CTNF@CoS_2_–CNA/S composite after cycling at 1 C for 100 times at both 25 and 55 °C was further characterized. As observed in Fig. S11, CTNF@CoS_2_–CNA/S cathode still maintained a good structural integrity as well as residual CNTs easily observed after cycling. Notably, compared to the fresh electrode, the surface of nitrogen-rich 3D conductive skeleton turned to be rough and dense, along with the CoS_2_/carbon nanoleaf arrays integrity was broken after cycling. The change in the morphology of CTNF@CoS_2_–CNA/S electrode after cycling can be ascribed to uniform SEI film formed and the accumulation of insoluble polysulfides, which could further be coated on the surface of foam during repeated charging and discharging process.

**Table 1:** Comparison of electrochemical performance with previously reported sulfur-based cathodes in LSBs at high temperature

Electrode materials	Capacity at different rates (mAh g^−1^)			Capacity after cycling (mAh g^−1^)	Capacity retention (%)	Sulfur (mg cm^−2^)	*T* (°C)	Refs.
	Low rate	1 C	2 C					
PGS-1000	220 (0.5 C)	200	150	1 C; 450	81	/	60	[49]
BN/graphene	1100 (0.5 C)	/	/	1 C; 1050	> 90	1.5	55	[50]
Bp2000-10	/	/	/	0.1 C; 1010	89	/	55	[51]
S/carbon black	/	/	/	0.2 C; 530 (0.125 C; 310)	54 (31%)	1.7–2.3	40 (60)	[52]
S@Co_3_S_4_ nanobox	446 (0.5 C)	/	/	0.2 C; 718	75.3	1.2	50	[53]
Sulfur–carbon composite	/	/	/	0.1 C; 250	16.1	1.75	45	[54]
CTNF@CoS_2_–CNA/S	1126 (0.5 C)	1013	881	1 C; 750	87	2.91 (3.47)	55	This work

Considering the outstanding mechanical properties and excellent conductivity of CTNF@CoS_2_–CNA configuration, it can be directly used as assemble foldable LSBs. Therefore, flexible soft-packaged LSBs on the basis of a sandwich-structured model were fabricated by using CTNF@CoS_2_–CNA/S composite as cathode, metallic Li foil as anode, together with Celgard 2400 as separator, finally sealed in an Al plastic film with appropriate electrolyte. The schematic diagram was described at Fig. S12. A “Li” model consisting of seven commercial blue light-emitting diodes (LEDs) was lighted up by resultant soft-packed LSBs (Fig. S12c–f), demonstrating that fabricated soft package batteries were able to work normally when the circuit connected. Meanwhile, the soft-packed battery can still work normally even after bending at an angle of 180 and returning to 0, persuasively verifying the outstanding flexibility of the soft-packaged. Therefore, the features of high flexibility combined with the admirable conductivity endow CTNF@CoS_2_–CNA configuration with the ability to apply in various stretchable/bendable wearable electronic devices.

### Catalytic Conversion and Trapping of Polysulfides on the CTNF@CoS_2_–CNA Electrode

**Fig. 5:**
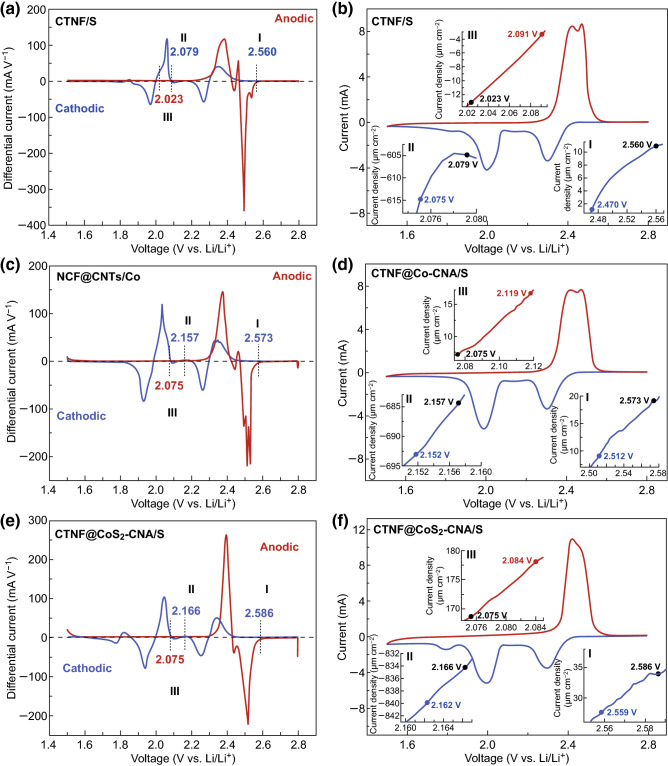
Electrocatalytic effects of electrode materials verified from the CV profiles: **a**, **c**, **e** differential CV curves and **b**, **d**, **f** CV curves of CTNF/S, CTNF@Co–CNA/S, and CTNF@CoS_2_–CNA/S electrodes. The corresponding onset potentials of redox peaks are provided in (**b**, **d**, **f**). The baseline potentials and baseline current densities in (**a, c, e**) are defined as the values before the redox peaks, where the variation on current density is the smallest, namely d*I*/d*V* = 0

The electrocatalytic effect of CTNF@CoS_2_–CNA was confirmed through the onset potential changes of three redox peaks (Fig. 5e, f). According to the common definition employed in electrocatalysis, the onset potentials were taken at a current density of 10 μA cm^−2^ beyond the baseline current. For comparation, the CTNF/S and CTNF@Co–CNA/S cathodes also tested to demonstrate the electrocatalytic effect (Fig. 5a–d). Compared with the two contrastive cathodes, the adoption of CTNF@CoS_2_–CNA/S cathode increased onset potentials of cathodic peaks and decreased onset potentials of anodic peak, which proving an accelerated electrocatalytic effect and elevated polysulfide redox kinetics [45]. Simultaneously, constructing novel cathode materials with high areal capacity and low self-discharge rate is particularly important. Hence, the self-discharge performance and cycling performance of three cathodes with high sulfur loading (~ 4.5 mg cm^−2^) were investigated. The open-circuit voltages (OCV) of three cathodes were recorded after 50 cycles at 0.2 C (Fig. S13), in which the downtrend of OCV for CTNF@CoS_2_–CNA/S cathode was slower than those of other two cathodes, indicating andante self-discharge condition of CTNF@CoS_2_–CNA. After fully charged and rest for 10 days, the capacity loss at the 51th discharge step of CTNF@CoS_2_–CNA/S was less than CTNF/S and CTNF@Co–CNA/S, suggesting that the CTNF@CoS_2_–CNA can efficiently alleviate the self-discharge behavior of LSBs.

To better reveal the catalytic capability of the CTNF@CoS_2_–CNA support on the electrochemical conversion of lithium polysulfides, the kinetics of polysulfide reduction and oxidation reactions in the liquid phase were studied on CTNF@CoS_2_–CNA as identical working and counter electrodes in a configured symmetric cell with the electrolyte containing 0.12 M Li_2_S_6_ in DOL/DME between − 1.4 and 1.4 V (Fig. S14). Symmetric cells can be directly used as a tester of polysulfides conversion in our electrode without the interference of lithium metal anode [46, 47]. Again, CTNF@Co–CNA and CTNF were assigned as the control samples. As shown, there are two pairs of redox peaks located at − 0.18/0.52 V and 0.18/− 0.52 V in the CV curves under 3 mV s^−1^ scan rate, corresponding to the reduction of sulfur to the polysulfides and then polysulfides to Li_2_S_2_/Li_2_S, respectively. These peaks appear sharp with narrow separation when the CTNF@Co–CNA electrode was used, indicating improved electrochemical reversibility and facile polysulfide conversion. Note that the Li_2_S_6_ symmetrical cell with the CTNF@CoS_2_–CNA/S electrodes showed a higher current response than its counterparts, further verifying that CoS_2_ as an efficient electrocatalysts can significantly generate faster reaction rates and enhanced kinetics of the soluble polysulfide redox reactions.

**Fig. 6:**
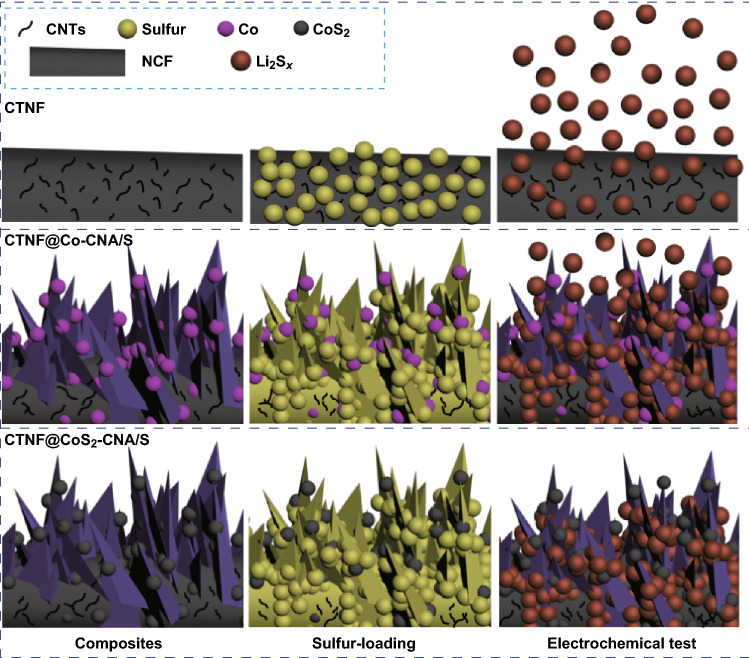
Schematic illustration of adsorption effects associated with “physical block and chemical absorption” for polysulfides based on CTNF, CTNF@Co–CNA, and CTNF@CoS_2_–CNA hybrids

According to the above experimental results and analysis, the hierarchical multifunctional architecture of CTNF@CoS_2_–CNA/S and its counter electrodes adsorbed with Li_2_S_*x*_ were schematically illustrated in Fig. 6. At the first discharge process, the soluble polysulfides can freely dissolve into the electrolyte if there is no adsorption medium. The CTNF@Co–CNA and CTNF electrodes keep part of the polysulfides inside the pores but cannot stop them from escaping from the surface layer. On the contrary, with the strong adsorbing effect of polar CoS_2_/carbon nanoleaf arrays, CTNF@CoS_2_–CNA hybrid can effectively absorb a large proportion of polysulfides by virtue of the polar–polar interaction, which contribute to preventing the shutting effect of polysulfides and reducing the loss of active materials, thus improving the electrochemical performance of LSBs significantly.

**Fig. 7:**
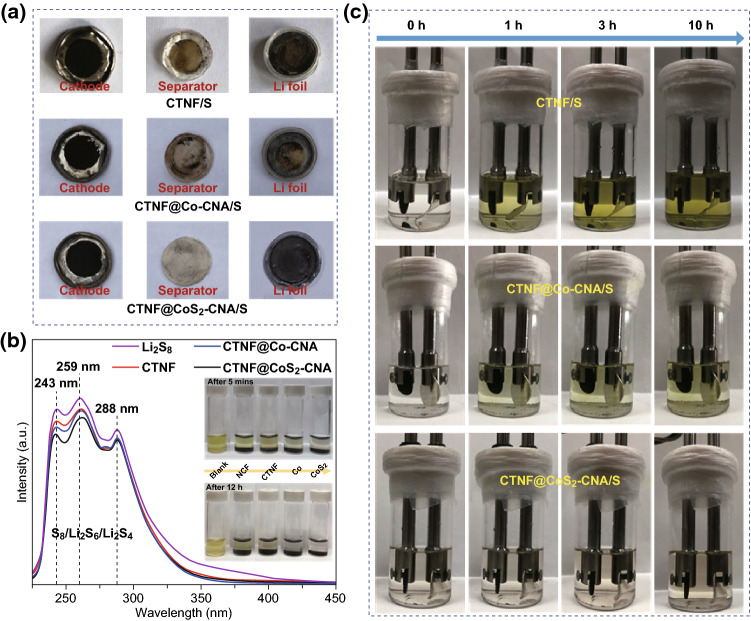
**a** Digital photographs of CTNF/S, CTNF@Co–CNA/S, and CTNF@CoS_2_–CNA/S cells after cycling at 1 C. **b** UV–Vis absorption spectra of various Li_2_S_8_ solutions, and the insets show the optical photographs of Li_2_S_8_ solutions before and after 12 h contact with the NCF, CTNF, CTNF@Co–CNA, and CTNF@CoS_2_–CNA materials, respectively. **c** Beaker cell tests of three cathodes

To explore the enhancement mechanism of the electrochemical properties of CTNF@CoS_2_–CNA cathode, we further executed postmortem analyses of cycled cells (Fig. 7a). Despite the cathodic integrity of assembled CTNF/S, CTNF@Co–CNA/S and CTNF@CoS_2_–CNA/S cells, an obvious severe anodic corrosion on the surface of lithium foil and the yellow-colored separator membrane surface for CTNF/S and CTNF@Co–CNA/S cells can be observed, indicating that the two cathodes suffered from firsthand pollution by soluble polysulfides in the electrolyte. Particularly for CTNF/S, it suffered more grievous corrosion and polysulfide shuttle. By contrast, the CTNF@CoS_2_–CNA/S cell exerted mild anodic corrosive situation along with relatively light-colored separator membrane, implying that there was no distinct soluble polysulfides contamination in the CTNF@CoS_2_–CNA/S cathode, since the strong absorption ability of CTNF@CoS_2_–CNA for the polysulfides. Besides, the same mass of NCF, CTNF, CTNF@Co–CNA, and CTNF@CoS_2_–CNA powder was added into the as-prepared Li_2_S_8_ solution for further visible adsorption capacity tests. The inset of Fig. 7b shows the degree of color change in the Li_2_S_8_ solution from NCF to CTNF@CoS_2_–CNA after contacting about 5 min and 12 h. Clearly, these four electrode materials exhibit a different adsorption capacity to S_8_^2−^ species. Compared with other samples, the Li_2_S_8_ solution immersed with CTNF@CoS_2_–CNA gradually became colorless, proving this material has superb trapping ability for high-order polysulfides. Based on this case, UV–Vis absorption spectra tests of CTNF, CTNF@Co–CNA, and CTNF@CoS_2_–CNA were further collected to investigate the adsorption ability for soluble polysulfides. Similar to adsorption test results, all the three samples displayed evident light absorption peaks located at the same position around 243, 257, and 288 nm in the UV–Vis absorption spectra, respectively; the wavelength coverage between 200 and 350 nm is attributed to a high degree of S_8_^2−^ ions [48]. Furthermore, original Li_2_S_8_ solution without any adsorbents exhibited darker color and the strongest absorbance. And due to the existence of in-suit nitrogen-doped and multiwalled CNTs-wrapped layer, NCF and CTNF exerted weak adsorption of polysulfides according to the UV–Vis absorption spectra. The solution soaked with CTNF@CoS_2_–CNA composite showed much weaker light absorption peaks, revealing its relatively strong chemical adsorption capacity to polysulfide anions and further efficiently trapping them around the cathode, which is consistent with previously reported literatures. Concurrently, beaker cell tests were completed to ulteriorly comprehend the exceptional ability of CTNF@CoS_2_–CNA for trapping soluble polysulfides. Figure 7c recorded the color changes of beaker cells at different time during discharging. CTNF/S, CTNF@Co–CNA/S, and CTNF@CoS_2_–CNA/S cathodes with same sulfur content were sealed at beakers, and the lithium foils as anodes were also encapsulated inside. Beaker cells were discharged at 0.1 C after injecting a certain amount of electrolyte. The electrolyte color quickly turned into deep yellow from colorless in the beaker based on CTNF/S cathode, manifesting the solvation and seepage of abundant lithium polysulfides into the electrolyte. For the CTNF@Co–CNA/S cathode, exerting good adsorption capacity to polysulfides, which proved through the electrolyte color gradually changed from colorless to canary yellow during the discharge process. Note that the electrolyte in the beaker based on CTNF@CoS_2_–CNA/S cathode remained colorless in the whole discharge process, indicating a little part of lithium polysulfides were dissolved in the electrolyte, which further demonstrate the remarkable polysulfide trapping capability of CTNF@CoS_2_–CNA composite. The outstanding adsorption capacity greatly improves the utilization of sulfur and enhances electrochemical properties of LSBs.

## Conclusion

In summary, our work has successfully fabricated a unique cobalt sulfide/carbon nanoleaf arrays rooting on N-rich 3D conductive carbon skeleton through self-assembly and vulcanized strategy. Benefiting from flexible 3D conductive network and MOF-derived CoS_2_/carbon nanoarrays, CTNF@CoS_2_–CNA hybrid possessed effective physisorption and chemisorption dual function for lithium polysulfides during electrochemical reaction. In addition, an accelerated electrocatalytic effect and improved polysulfide redox kinetics arising from CoS_2_–CNA also stimulated the excellent rate capacity of CTNF@CoS_2_–CNA/S electrode. Thus, the CTNF@CoS_2_–CNA/S electrode exhibits outstanding rate performance of 1499 mAh g^−1^ at 0.1 C and 881 mAh g^−1^ at 2 C with the sulfur loading of 2.91 mg cm^−2^ even operated at 55 °C. More remarkably, with the ultrahigh sulfur loading of 7.19 mg cm^−2^, the CTNF@CoS_2_–CNA/S cathode still exhibits high rate capacity (602 mAh g^−1^, 0.5 C; 450 mAh g^−1^, 1 C). Furthermore, the as-prepared CTNF@CoS_2_–CNA/S material also can be directly used as soft package cathode to apply in various stretchable/bendable electronic devices. More importantly, the reasonable cathode design scheme can easily yield an integrated overall advantage and further exhibit great potential for various flexible/wearable energy storage applications at different temperature environment.

## Electronic supplementary material

Below is the link to the electronic supplementary material.

Supplementary material 1 (PDF 900 kb)
