# Supplementary material for: Construction of Electrocatalytic and Heat-Resistant Self-Supporting Electrodes for High-Performance Lithium–Sulfur Batteries
Source: Nanomicro Lett. 2019 Sep 18;11:78. doi: 10.1007/s40820-019-0313-x (PMC7770752; doi:10.1007/s40820-019-0313-x)
Supplement: Supplementary file 1 — Supplementary material 1 (PDF 900 kb) [file 40820_2019_313_MOESM1_ESM.pdf]

Supporting Information for

Construction of Electrocatalytic and Heat-Resistance Self-Supporting Electrodes for High-Performance Lithium-Sulfur Batteries

Xuemei Zhang[1, +], Yunhong Wei[1, +], Boya Wang[2], Mei Wang[2], Yun Zhang[2, *], Qian Wang [2], Hao Wu[1, *]

[1]College of Materials Science and Engineering, Sichuan University, Chengdu 610064, People's Republic of China

[2]Department of Advanced Energy Materials, Sichuan University, Chengdu 610064,People's Republic of China

[+]These authors contributed equally to this work

*Corresponding authors. E-mail: y_zhang@scu.edu.cn (Yun Zhang); hao.wu@scu.edu.cn (Hao Wu)

Supplementary Figures and Tables

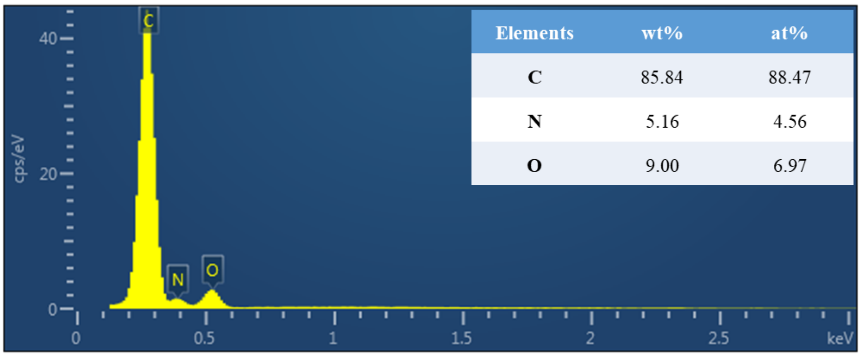

Elements	wt%	at%
C	85.84	88.47
N	5.16	4.56
O	9.00	6.97

Fig. S1 EDX spectra of NCF

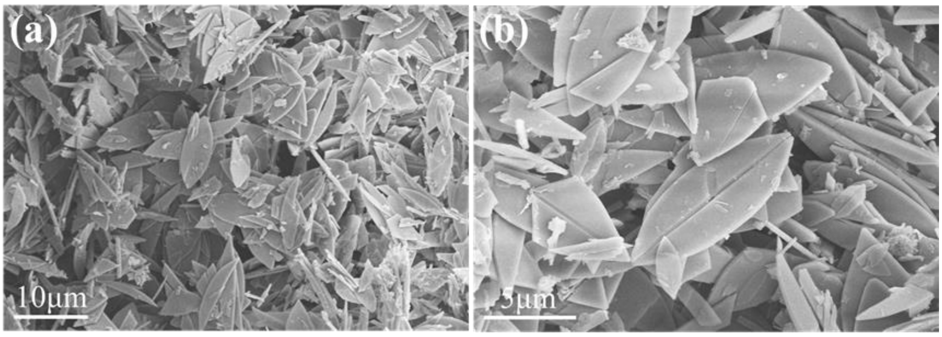

Fig. S2 SEM images of ZIF-67 precursor

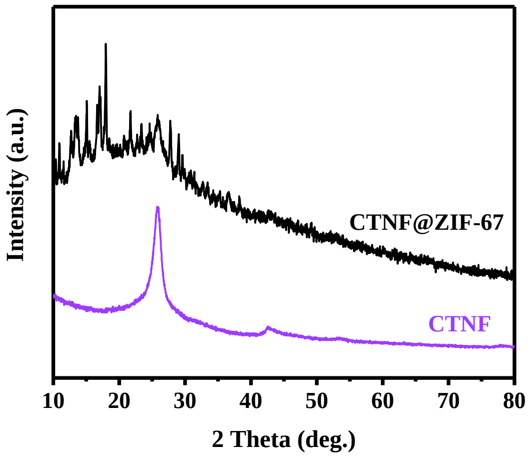

Fig. S3 XRD patterns of simulated CTNF and CTNF/ZIF-67

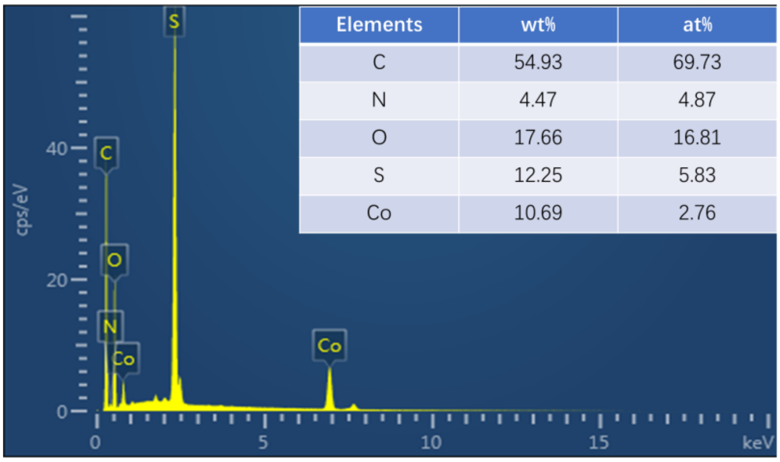

Fig. S4 EDX spectra of CTNF/CoS_2-CNA

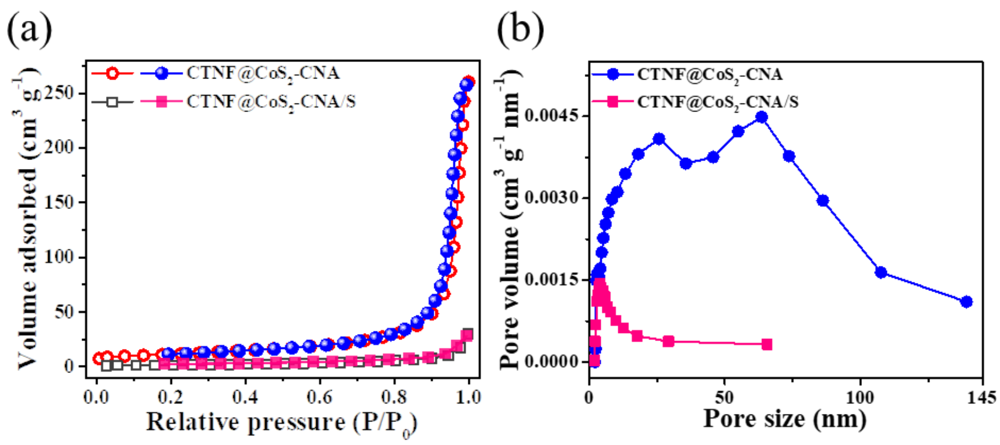

Fig. S5 a N_2 adsorption−desorption isotherm curve and **b** Pore size distribution of CTNF@CoS_2-CNA and CTNF@CoS_2-CNA/S (~3 mg cm^{-2})

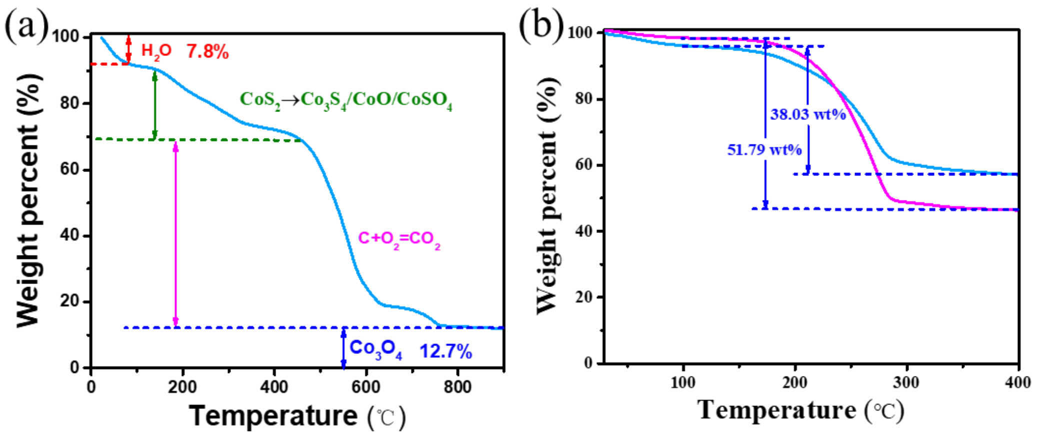

Fig. S6 TGA curves recorded for **a** CTNF@CoS_2-CNA under air flow with the heating rate 10 °C min^{-1}; **b** CTNF@CoS_2-CNA/S under nitrogen flow with the heating rate 10 °C min^{-1}

In addition to the stage at below 100°C caused by the evaporation of H_2O, there are two tages of weight loss for CTNF@CoS_2-CNA. The first stage at below 450 °C is attributed to the oxidation of CoS_2 in the composite [S1, S2]. This is due to the complex reactions including the formation of Co_3S_4, CoO and $CoSO_4$ intermediate products accompanied with the oxidation of carbon. The second continuous weight loss in the range of 450-760 °C results from the combustion of total carbon (CNT, CF, and MOF-derived carbon) in CTNF/CoS_2-CNA. Nevertheless, all the intermediates would transform into Co_3O_4 as the temperature increased to 850 °C [S3]. Thus, the total reaction can be simply written as Eqs. S1 and S2:

$$3CoS_{2\,(s)}+8O_{2\,(g)}=Co_3O_{4\,(s)}+6SO_{2\,(g)} \qquad (S1)$$

$$C_{(s)}+O_{2\,(g)}=CO_{2\,(g)} \qquad (S2)$$

According to the weight of the Co_3O_4, it can be calculated the weight of the CoS_2. Eliminating the effect of H_2O, the mass content of Co_3O_4 is calculated as 12.7/(100%-7.8%)=13.8%. Therefore, on the basis of the reaction (S1), the mass content of CoS_2 in CTNF/CoS_2-CNA is around 20.9%.

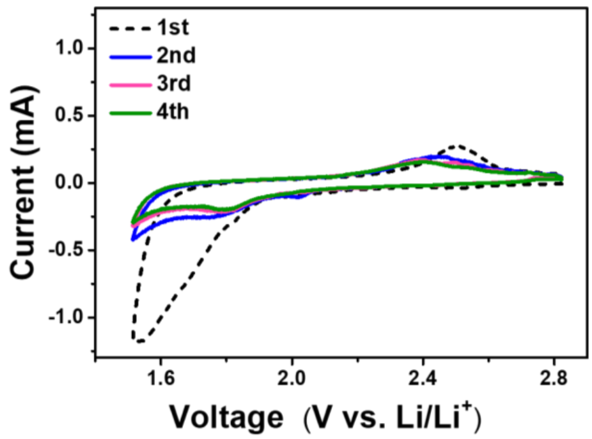

Fig. S7 CV curve of CTNF@CoS_2-CNA matrix

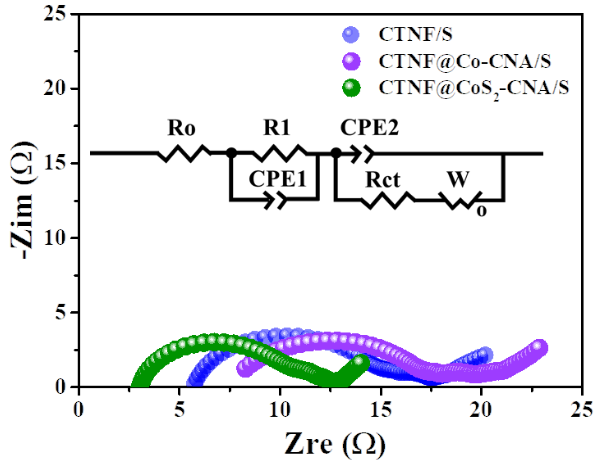

Fig. S8 Comparison of electrochemical impedance spectra after 100 charge-discharge cycles at room temperature

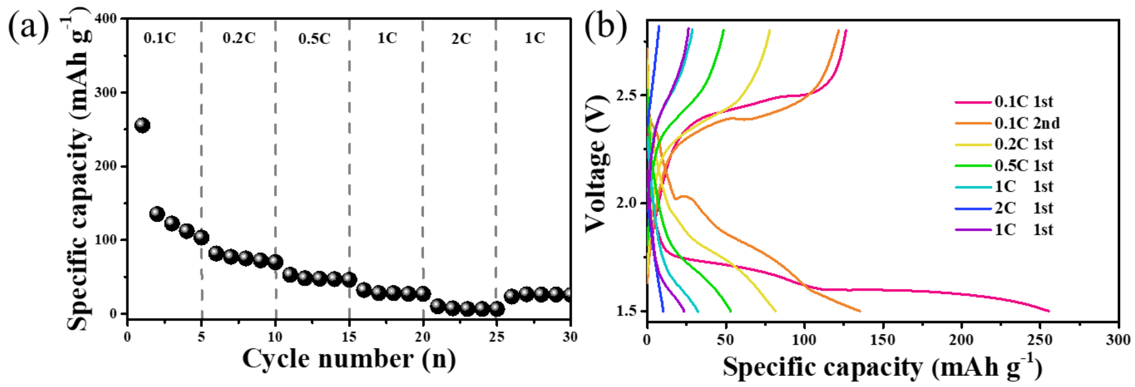

Fig. S9 a Rate performance from 0.1 C to 2 C at room temperature; **b** discharge/charge profiles at different rates

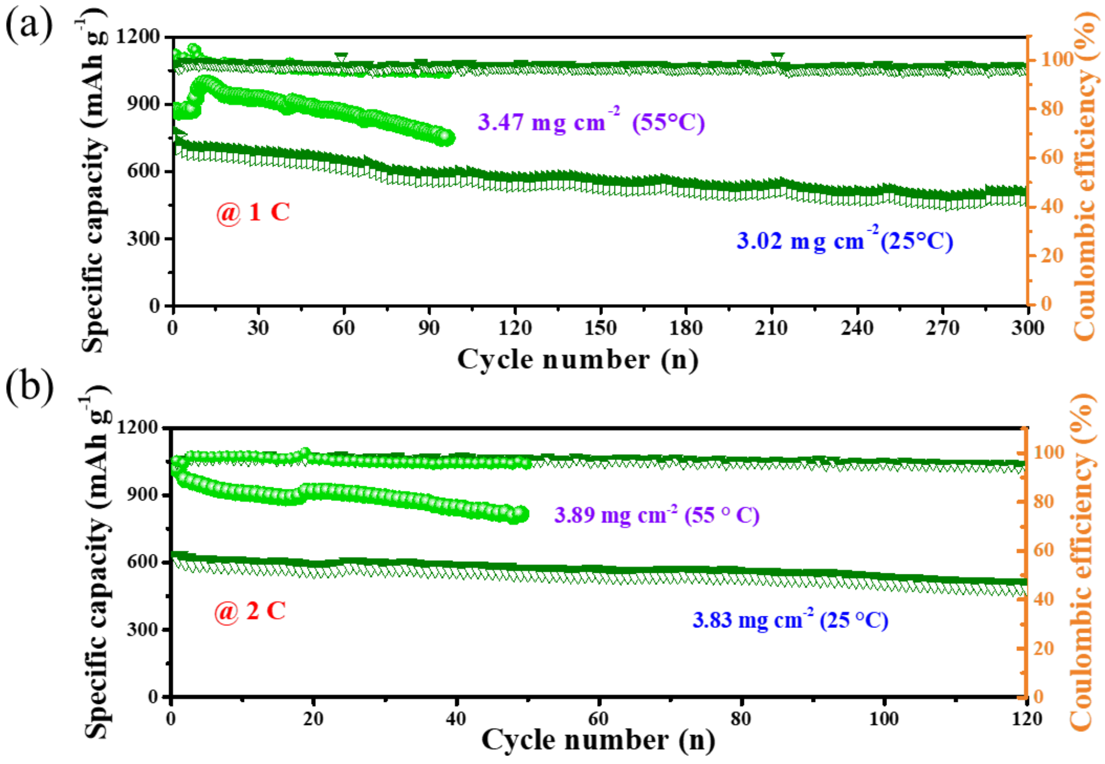

Fig. S10 Cycling performance comparison of CTNF@CoS_2-CNA/S tested at room/high temperature with different current density: **a** 1 C and **b** 2 C

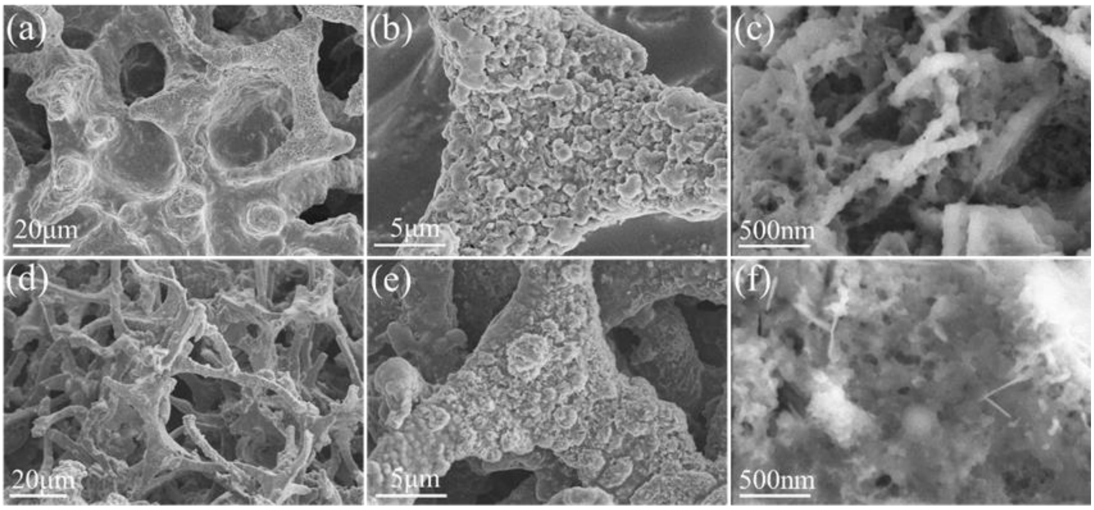

Fig. S11 SEM images of CTNF@CoS_2-CNA/S electrode after cycled at different temperature: **a-c** room temperature and **d-f** high temperature

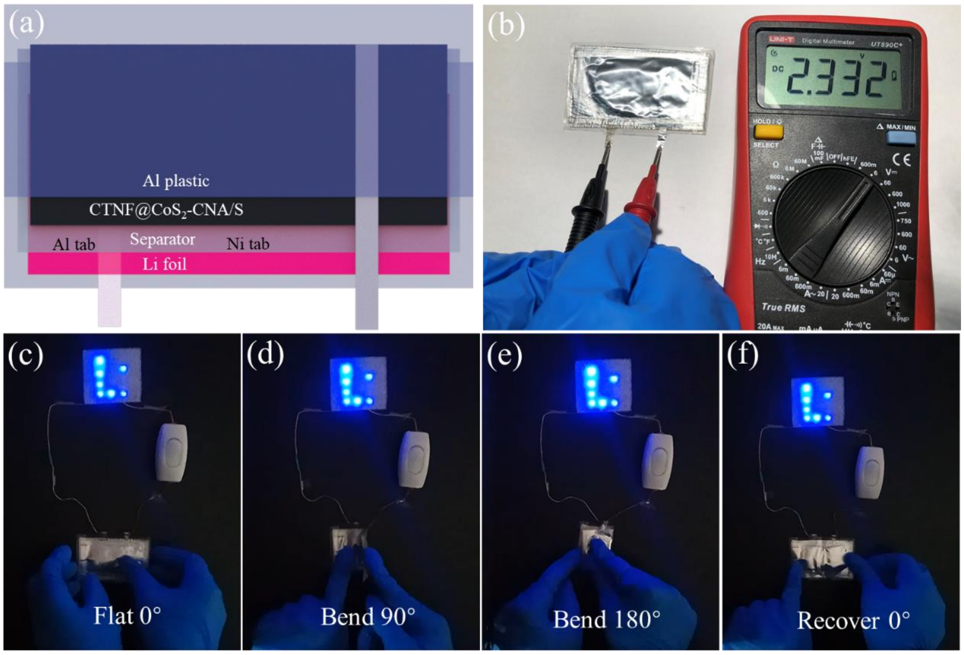

Fig. S12 a Schematic diagram of soft-packed CTNF@CoS_2-CNA/S battery; **b** Photograph of the soft-packed battery at charged state; **c-f** The CTNF@CoS_2-CNA/S battery used to light "Li" model LEDs after bending at 0°, 90°, 180°, and recover to 0°

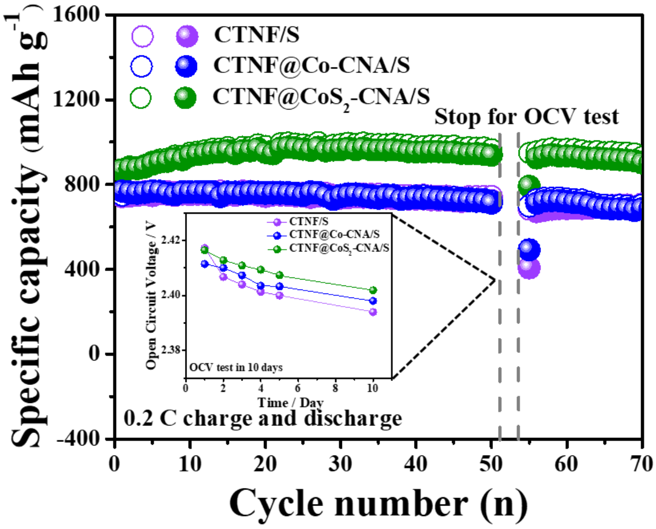

Fig. S13 Self-discharge rate tests of three electrodes with the standing time of 10 days after the 50 cycles at 0.2 C

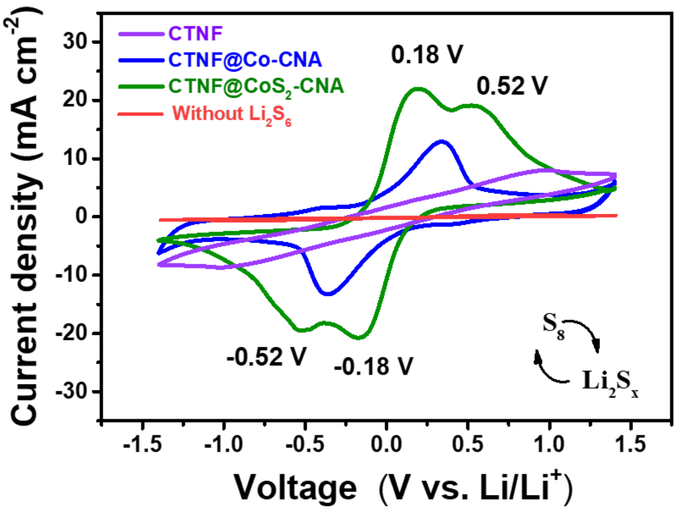

Fig. S14 Catalytic effects of electrode materials on the lithium polysulfide conversion: CV curves of symmetric cells of CTNF, CTNF@Co-CNA and CTNF@CoS_2-CNA electrodes in the electrolyte with 0.12 M Li_2S_6 at scan rate of 3 mV s^{-1}

Table S1 The electrical conductivity of the samples

Samples	Compounds	Electronic conductivity (S cm^{-1})
Without sulfur	NCF	0.0011
	CTNF	0.0517
	CTNF@Co-CNA	0.0837
	CTNF@CoS_2-CNA	0.1596
With sulfur	CTNF@ Co-CAN/S	0.0365
	CTNF@ CoS_2-CAN/S	0.0571

Table S2 Comparison of electrical conductivity with previously reported sulfur-based cathodes in LSBs

Materials	Compound	Electronic conductivity (S cm^{-1})	Refs.
Metal oxides or carbonaceous metal oxides	Fe_2O_3	2.2×10^{-6}	[S4]
	Fe_2O_3-graphene	0.156	[S5]
	NiO-GNS	1.4×10^{-3}	[S6]
Metal sulfides or carbonaceous metal sulfides	SnS_2	1.0×10^{-3}	[S4]
	SnS_2-RGO	0.037	[S7]
	CoS_2/RGO-CNT	7.2×10^{-4}	[S8]
	CTNF@CoS_2-CNA	0.1596	This work

Table S3 The porous structure parameters of the CTNF@CoS_2-CNAand CTNF@CoS_2-CNA/S

Samples	BET surface area (m^2 g^{-1})	Pore volume (cm^3 g^{-1})	Average pore size (nm)
CTNF@CoS_2-CNA	39.6706	0.4025	20.29
CTNF@CoS_2-CNA/S	5.1812	0.0465	17.95

Table S4 The R_{ct} values of cells before and after cycling according to equivalent circuit fitting

Samples	CTNF	CTNF@Co-CNA	CTNF@CoS_2-CNA
Fresh cell	77.83	53.2	29.21
After cycling	9.67	8.77	6.78

Table S5 Comparison of electrochemical performance with previously reported sulfur-based cathodes in LSBs at room temperature

Electrode materials	Capacity at different rates (mAh g^{-1})			Capacity after cycling (mAh g^{-1})	Cycles	Sulfur (mg cm^{-2})	Refs.
	Low rate	1 C	2 C				
Carbon/S	750 (0.5 C)	600	400	1 C; 662	100	1.8	[S9]
HC-TiO_2/S	1050 (0.1 C)	716	621	1 C; 691	300	1.2-1.8	[S10]
CeO_2/MMNC-S	980 (0.2 C)	680	520	0.5 C; 650	200	3.4	[S11]
50%-MWCNTs@TiO_2-S	900 (0.1 C)	360	300	0.1 C; 679	50	2.0	[S12]
S@C@MnO_2	1080 (0.5 C)	985	800	0.5 C; 712	300	3.0	[S13]
ICFs/nS/rGO	950 (0.2 C)	/	/	0.1 C; 892	200	2.8	[S14]
rGO/ppy/S	900 (0.5 C)	828	747	1 C; 770	100	1.32	[S15]
ANC/S-70	970 (0.2 C)	800	750	1 C; 700	500	1.0	[S16]
CTNF@CoS_2-CNA/S	993 (0.2 C)	805	698	1 C; 851	100	3.48 (3.10)	This work

Table S6 Comparison of the electrochemical performance of previously reported cobalt sulfide nanocomposite electrode materials with our work

Sulfur host materials	Areal mass loading (mg cm^{-2})	Current rate /Capacity (mAh g^{-1})		Cycle number	Cycle capacity (mAh g^{-1})	Refs.
		0.1 C	2 C			
CTNF@CoS2-C NA	3.02	1029	698	300	505 (1 C)	This work
Co_9S_8/C nanopolyhedra	3.0	950	/	200	790 (0.5 C)	[S17]
CoS_2-NC	1.3	1060	708	250	600 (1 C)	[S18]
Co_3S_4 nanotubes	Wt:79.3%	1040	608	200	815 (0.5 C)	[S19]
S/GN–CNT composite	1.3–1.6	1045	408	500	363 (1 C)	[S20]
CoS@PPy/S	1.4–1.6	1000 (0.2 C)	536	500	700 (0.2 C)	[S21]
graphene-like Co_9S_8	1.5	/	863	400	512 (0.5 C)	[S22]

Supplementary References

[S1]Z. Chen, R. Wu, M. Liu, H. Wang, H. Xu et al., General synthesis of dual carbon-confined metal sulfides quantum dots toward high-performance anodes for sodium-ion batteries. Adv. Funct. Mater. **27**(38), 1702046 (2017). https://doi.org/10.1002/adfm.201702046

[S2]C. Xu, Y. Jing, J. He, K. Zhou, Y. Chen, Q. Li, J. Lin, W. Zhang, Self-assembled interwoven CoS_2/CNTs/graphene architecture as anode for high-performance lithium ion batteries. J. Alloy. Compd. **708**, 1178-1183 (2017). https://doi.org/10.1016/j.jallcom.2017.03.099

[S3]B.Y. Xia, Y. Yan, N. Li, H.B. Wu, X.W. Lou, X. Wang, A metal-organic framework-derived bifunctional oxygen electrocatalyst. Nat. Energy **1**(1), 15006 (2016). https://doi.org/10.1038/nenergy.2015.6

[S4]Y. Zhao, L.P. Wang, M.T. Sougrati, Z. Feng, Y. Leconte, A. Fisher, M. Srinivasan, Z. Xu, A review on design strategies for carbon based metal oxides and sulfides nanocomposites for high performance Li and Na ion battery anodes. Adv. Energy Mater. **7**(9), 1601424 (2017). https://doi.org/10.1002/aenm.201601424

[S5]J. Kan, Y. Wang, Large and fast reversible Li-ion storages in Fe_2O_3-graphene sheet-on-sheet sandwich-like nanocomposites. Sci. Rep. **3**, 3502 (2013). https://doi.org/10.1038/srep03502

[S6]Y. Zou, Y. Wang, NiO nanosheets grown on graphene nanosheets as superior anode materials for Li-ion batteries. Nanoscale **3**(6), 2615-2620 (2011). https://doi.org/10.1039/c1nr10070j

[S7]P. Chen, Y. Su, H. Liu, Y. Wang, Interconnected tin disulfide nanosheets grown on graphene for Li-ion storage and photocatalytic applications. ACS Appl. Mater. Interfaces **5**(22), 12073-12082 (2013). https://doi.org/10.1021/am403905x

[S8]S. Peng, L. Li, X. Han, W. Sun, M. Srinivasan et al., Cobalt sulfide nanosheet/graphene/carbon nanotube nanocomposites as flexible electrodes for hydrogen evolution. Angew. Chem. Int. Ed. **53**(46), 12594-12599 (2014). https://doi.org/10.1002/anie.201408876

[S9]J. Wu, J. Hu, K. Song, J. Xu, H. Gao, Spirulina-derived nitrogen-doped porous carbon as carbon/S composite cathodes for high cyclability lithium-sulphur batteries. J. Alloy. Compd. **704**, 1-6 (2017). https://doi.org/10.1016/j.jallcom.2017.02.052

[S10]J. Yao, T. Mei, Z. Cui, Z. Yu, K. Xu, X. Wang, Hollow carbon spheres with TiO2 encapsulated sulfur and polysulfides for long-cycle lithium-sulfur batteries. Chem. Eng. J. **330**, 644-650 (2017). https://doi.org/10.1016/j.cej.2017.08.006

[S11]L. Ma, R. Chen, G. Zhu, Y. Hu, Y. Wang, T. Chen, J. Liu, Z. Jin, Cerium oxide nanocrystal embedded bimodal micromesoporous nitrogen-rich carbon nanospheres as effective sulfur host for lithium-sulfur batteries. ACS Nano **11**(7), 7274-7283 (2017). https://doi.org/10.1021/acsnano.7b03227

[S12]X. He, H. Hou, X. Yuan, L. Huang, J. Hu et al., Electrocatalytic activity of lithium polysulfides adsorbed into porous TiO_2 coated MWCNTs hybrid structure for lithium-sulfur batteries. Sci. Rep. **7**, 40679 (2017). https://doi.org/10.1038/srep40679

[S13]L. Ni, G. Zhao, G. Yang, G. Niu, M. Chen, G. Diao, Dual core-shell-structured $S@C@MnO_2$ nanocomposite for highly stable lithium-sulfur batteries. ACS Appl. Mater. Interfaces **9**(40), 34793-34803 (2017). https://doi.org/10.1021/acsami.7b07996

[S14]F. Wu, Y.S. Ye, J.Q. Huang, T. Zhao, J. Qian et al., Sulfur nanodots stitched in 2D "Bubble-Like" interconnected carbon fabric as reversibility-enhanced cathodes for lithium-sulfur batteries. ACS Nano **11**(5), 4694-4702 (2017). https://doi.org/10.1021/acsnano.7b00596

[S15]W. Qian, Q. Gao, H. Zhang, W. Tian, Z. Li, Y. Tan, Crosslinked polypyrrole grafted reduced graphene oxide-sulfur nanocomposite cathode for high performance Li-S Battery. Electrochim. Acta **235**, 32-41 (2017). https://doi.org/10.1016/j.electacta.2017.03.063

[S16]X. Wu, L. Fan, M. Wang, J. Cheng, H. Wu, B. Guan, N. Zhang, K. Sun, Long-life lithium-sulfur battery derived from nori-based nitrogen and oxygen dual-doped 3D hierarchical biochar. ACS Appl. Mater. Interfaces **9**(22), 18889-18896 (2017). https://doi.org/10.1021/acsami.7b04583

[S17]T. Chen, L. Ma, B. Cheng, R. Chen, Y. Hu et al., Metallic and polar Co_9S_8 inlaid carbon hollow nanopolyhedra as efficient polysulfide mediator for lithium−sulfur batteries. Nano Energy **38**, 239-248 (2017). https://doi.org/10.1016/j.nanoen.2017.05.064

[S18]J. Zhou, N. Lin, W.l. Cai, C. Guo, K. Zhang, J. Zhou, Y. Zhu, Y. Qian, Synthesis of S/CoS_2 nanoparticles-embedded N-doped carbon polyhedrons from polyhedrons ZIF-67 and their properties in lithium-sulfur batteries. Electrochim. Acta **218**, 243-251 (2016). https://doi.org/10.1016/j.electacta.2016.09.130

[S19]J. Pu, Z. Shen, J. Zheng, W. Wu, C. Zhu et al., Multifunctional Co_3S_4@sulfur nanotubes for enhanced lithium-sulfur battery performance. Nano Energy **37**, 7-14 (2017). https://doi.org/10.1016/j.nanoen.2017.05.009

[S20]Z. Zhang, L.-L. Kong, S. Liu, G.-R. Li, X.-P. Gao, A high-efficiency sulfur/carbon composite based on 3D graphene nanosheet@carbon nanotube matrix as cathode for lithium-sulfur battery. Adv. Energy Mater. **7**(11), 1602543 (2017). https://doi.org/10.1002/aenm.201602543

[S21]Y. Song, H. Wang, W. Yu, J. Wang, G. Liu et al., Synergistic stabilizing lithium sulfur battery via nanocoating polypyrrole on cobalt sulfide nanobox. J. Power Sources **405**, 51-60 (2018). https://doi.org/10.1016/j.jpowsour.2018.10.032

[S22]Z.W. Seh, J.H. Yu, W. Li, P.C. Hsu, H. Wang et al., Two-dimensional layered transition metal disulphides for effective encapsulation of high-capacity lithium sulphide cathodes. Nat. Commun. **5**, 5017 (2014). https://doi.org/10.1038/ncomms6017
